# Bitter taste receptors are involved in asthma relieved by aspirin: A hypothesis 

**DOI:** 10.5414/ALX400683

**Published:** 2026-10-30

**Authors:** César Picado, Miguel Perpiñá

**Affiliations:** 1 Hospital Clinic, School of Medicine and Health Sciences, University of Barcelona, Barcelona,; 2 Centro de Investigationes Biomédicas en Red de Enfermedades Respiratorias (CIBERES), Madrid, and; 3 Respiratory Department, Hospital Universitario La Fé, Valéncia, Spain

**Keywords:** aspirin, asthma, cyclooxygenase, NSAIDS, taste type 2 receptors, bitter taste receptor

## Abstract

Nonsteroidal anti-inflammatory drugs (NSAIDs) typically do not affect asthma symptoms in most people. However, there are unique subgroups of patients whose responses to NSAIDs diverge considerably from the norm. Among these individuals, some experience an exacerbation of their asthma – commonly identified as aspirin- or NSAID-exacerbated respiratory disease (AERD and N-ERD, respectively). In contrast, others exhibit a remarkable improvement in their asthma symptoms following the administration of NSAIDs. This improvement is characterized by rapid, pronounced, and long-lasting bronchodilation, resulting in a substantial improvement in clinical symptoms and enhanced respiratory function. The observation of such contrasting responses has prompted further investigation into the mechanisms that might explain this phenomenon. Our hypothesis centers on the activation of bitter taste receptors by NSAIDs, resulting in rapid, significant, and sustained bronchodilation. Notably, this bronchodilator effect closely mirrors the relief observed in those asthma cases that improve with NSAID use. This mechanism provides a potential biological basis for their distinct response to NSAID therapy.

## Introduction 

Nonsteroidal anti-inflammatory drugs (NSAIDs) generally do not affect asthma symptoms for most users. However, some individuals experience serious symptom flare-ups – a condition called NSAID-exacerbated respiratory disease (N-ERD), also known as aspirin-exacerbated respiratory disease (AERD) – often linked to moderate to severe asthma and chronic rhinosinusitis with nasal polyps (CRSwNP) [1]. Surprisingly, there is a small group of patients for whom NSAIDs provide pronounced relief from asthma symptoms. In these cases, the drugs induce rapid and potent bronchodilation, resulting in a significant improvement in respiratory function [2, 3, 4, 5, 6, 7, 8, 9, 10]. 

In most documented cases where asthma symptoms improved following NSAID administration, the onset of asthma typically occurred during middle age and affected both males and females. Prior to the development of asthma, some patients experienced persistent rhinorrhea and nasal congestion. Among those evaluated for comorbid conditions, chronic sinusitis was diagnosed in more than half of the patients. Prick testing for common allergens yielded negative results in all patients tested [2, 3, 4, 5, 6, 7, 8, 9, 10]. There have also been isolated reports of patients with CRSwNP who, upon receiving NSAIDs, experienced relief from nasal symptoms. These improvements included restoration of the sense of smell and a reduction in the size of nasal polyps [11]. 

The frequency of asthma relieved by NSAIDs has been reported in studies using different populations and methods. Richard S.B. Pearson was a pioneering clinical immunologist whose work in the 1960s highlighted the clinical paradox regarding the relationship between aspirin and asthma. Pearson [12] reported that 6 patients out of 1,205 asthmatics reported that their symptoms were relieved or prevented by the drug. 

In another study, Resta et al. [13] administered 750 mg of intravenous aspirin to 100 asthmatic patients without N-ERD. Spirometry was performed at several time points after administration. 64 patients showed no change in lung function, 14 had less than 20% variation in forced expiratory volume in 1 second (FEV_1_), and 14 showed possible bronchodilation, defined as a 15 – 20% increase in FEV_1_. In patients whose FEV_1_ increased by 20%, the test was repeated; bronchodilation was confirmed in only 2 cases. 

Numerous NSAIDs have been assessed in blinded oral studies and compared with placebo and analgesics, such as paracetamol (acetaminophen), sodium salicylate, and benzydamine. The following NSAIDs have been tested in the studies: indomethacin (50, 75 mg), ibuprofen (400, 600 mg), dipyrone (575 mg), fenoprofen (400 mg), mefenamic acid (75 mg), noramidopyrine (500 mg), and ketoprofen (75 mg) [2, 3, 4, 5, 6, 7, 8, 9, 10]. A bronchodilator response was consistently observed following the administration of all NSAIDs tested in these studies, except paracetamol, which did not produce any significant bronchodilator effect [2, 3, 4, 5, 6, 7, 8, 9, 10]. 

All reported instances of asthma improvement following aspirin use have been based on self-reported patient experiences [2, 3, 4, 5, 6, 7, 8, 9, 10]. The reliance on patient reports and the scarcity of data on aspirin testing in the general population point to two possible explanations for this phenomenon. First, aspirin-relieved asthma may constitute a distinct and independent subgroup within the overall asthma population. Alternatively, this effect might represent just the visible portion of a broader spectrum, a “tip of the iceberg” scenario. In this view, only those patients experiencing the most pronounced benefits are likely to self-report their responses, whereas patients with mild or moderate effects may not recognize or attribute these improvements to aspirin. As a result, the milder responses go unnoticed and unreported, potentially being perceived as part of aspirin’s general contribution to overall well-being rather than as specific asthma relief. 

The way patients with aspirin-relieved asthma respond to challenges involving aspirin and other NSAIDs demonstrate several unique characteristics that distinguish this subgroup [2, 3, 4, 5, 6, 7, 8, 9, 10].

- There are currently no studies that systematically assess the dose-response relationship for aspirin in these patients, meaning the lowest and highest effective doses remain undetermined. All existing studies have used aspirin doses of at least 500 mg, and a maximum dose of 900 mg.
- The bronchodilator effect manifests rapidly, becoming detectable within 30 – 60 minutes following exposure to NSAIDs.
- Most patients demonstrate a significant enhancement in pulmonary function, with improvements generally exceeding 3 or more times those usually achieved with β-adrenergic agents such as salbutamol.
- Lung function continues to improve in the hours following administration. The duration of this response varies among individuals, but in some cases, pulmonary function remains enhanced for up to 20 hours after receiving aspirin.
- When aspirin is administered daily over a period of 7 days, the beneficial effect on lung function is consistently maintained.

## Discussion 

We first discuss the pros and cons of the hypothesis that arachidonic acid (AA) metabolism is involved in aspirin-relieved asthma, then those regarding our hypothesis that bitter taste receptor activation may explain this unusual effect of NSAIDs. 

### Role of the COX pathway in asthma improved by NSAIDs 

Current evidence indicates several defining features regarding how NSAIDs alleviate asthma symptoms. First, research supports the existence of a shared mechanism among all NSAIDs in producing this therapeutic effect. Second, the nature of the response – characterized by its rapid onset, progressive improvement, and sustained duration – suggests the direct activation of a specific receptor, rather than an indirect process, such as the gradual reduction of airway inflammation over time. Third, the responsiveness of this receptor is maintained even after repeated exposure to the agonist, indicating that receptor expression does not diminish with continued NSAID administration. As a result, patients continue to experience relief with successive doses, underscoring the stability of the mechanism involved. 

Both NSAID-relieved asthma and N-ERD are characterized by the ability of various NSAIDs to elicit a response in certain patients. This similarity has prompted researchers to suggest that the underlying mechanisms of NSAID-relieved asthma might somehow be linked to those driving N-ERD. The prevailing approach has been to examine whether these two conditions might share a common biological pathway but exert opposite effects, with the expectation that examining the mechanism of N-ERD will shed light on NSAID-relieved asthma [4, 5, 6, 7, 8, 9, 10, 14]. 

The mechanisms underlying N-ERD are partly understood. In individuals with N-ERD, there is a reduction in COX enzyme activity in AA metabolism, specifically COX-1, which is involved in normal physiological processes, and COX-2, which is typically upregulated during inflammatory conditions [15]. This decrease in COX-1 and COX-2 activity occurs in airway epithelial and fibroblast cells. As a result of diminished COX activity, the reduced production of prostaglandin E2 (PGE_2_) contributes to an increased baseline release of cysteinyl leukotrienes (Cys-LTs) and prostaglandin D2 (PGD_2_) from mast cells and eosinophils, which are potent bronchoconstrictor mediators [1, 16]. When NSAIDs are administered, they further suppress the synthesis of PGE_2_. This leads to a surge in Cys-LT production and a paradoxical increase in PGD_2_ release [16]. Both Cys-LTs and PGD_2_ exacerbate asthma symptoms and cause respiratory distress [1]. 

The EP2 receptor plays a key role in mediating the protective effects of PGE_2_; in N-ERD patients, EP2 receptor expression on inflammatory cells is decreased [17]. This decrease impairs the anti-inflammatory response, leading to ongoing airway inflammation [1, 17, 18]. 

Following an episode of aspirin-induced bronchoconstriction, there is typically a refractory period lasting from 2 to 4 days. During this time, patients may be able to use NSAIDs without experiencing respiratory symptoms. This timeframe mirrors the biological rate at which nucleated cells can produce new functional COX-1 enzymes [1, 19]. 

Based on the N-ERD mechanism, one hypothesis has been proposed to explain why some individuals experience improvement in asthma symptoms following the administration of NSAIDs. In these cases, it is suggested that AA metabolism is preferentially directed toward the COX pathway, leading to increased production of bronchoconstrictive prostaglandins, such as prostaglandin F_2_α (PGF_2_α) and PGD_2_. When NSAIDs are administered, they inhibit COX enzymes, which suppress the heightened basal production of these potent bronchoconstrictors. Consequently, bronchospasm relief is observed, leading to noticeable improvement in respiratory symptoms for affected patients [4, 7, 8, 10, 14]. However, this theory does not fully clarify how NSAIDs can induce very rapid and potent bronchodilators through an indirect mechanism that presumably requires time to develop. Additionally, the observation that repeated aspirin administration on consecutive days produces beneficial effects challenges this explanation. This is because aspirin’s irreversible inhibition of COX enzymes is typically associated with a refractory period during which further effects, either positive or negative, would not be expected. If the bronchodilator response were a result of COX-1 inhibition, it would be logical to expect that – like the bronchoconstrictor effect – the bronchodilator response would not recur until new COX-1 is synthesized. 

Another potential hypothesis involves lipoxins (LXs), specifically LXA_4_ and LXB_4_. These are produced from AA through transcellular metabolism involving the coordinated actions of neutrophils, platelets, epithelial cells, and the enzymes 15- and 5-lipoxygenases (LOX) [20]. An alternative pathway for LX synthesis is activated by aspirin-acetylated COX-2, leading to the formation of aspirin-triggered lipoxins (ATLs), known as 15R epimers or 15-epi-LXs. These are generated through endothelial-leukocyte interactions [20]. N-ERD patients have diminished capacity to generate LXA4 and its 15-epimer [21, 22]. 

LXs act as important anti-inflammatory agents within the respiratory system. They help protect against airway obstruction triggered by Cys-LTs – known mediators of bronchoconstriction. LXs achieved this anti-inflammatory protective effect through several mechanisms: inhibiting granulocyte activation and blocking the activity of the pro-inflammatory cytokines and chemokines that contribute to airway inflammation and narrowing [20]. 

Unlike agents that provide immediate bronchodilation, LXs act by stabilizing airway homeostasis and preventing excessive airway constriction over time. This regulatory role reduces the risk of acute airway narrowing and helps maintain normal respiratory function [20]. 

Clinical studies have demonstrated that low-dose aspirin therapy, typically 81 mg per day, is effective in promoting the synthesis of ATLs. This effect is unique to low-dose regimens and is not observed with higher aspirin doses, such as those commonly prescribed for the treatment of aspirin-relieved asthma. At these elevated therapeutic doses, there is no significant induction of ATL production. This dose-dependent phenomenon underscores the distinct biological response elicited by low-dose aspirin compared with higher doses [23]. 

The lack of ATL generation at higher therapeutic aspirin doses suggests that ATL production does not account for the bronchodilator effects seen in patients whose asthma symptoms are relieved by these medications. 

Investigations into the metabolic pathways of AA in platelets have been conducted to determine whether aspirin-responsive asthma patients exhibit any unique abnormalities. Specifically, two separate studies evaluated the AA metabolism in individuals who experience symptom relief from aspirin therapy. The findings from these studies revealed no significant differences in AA metabolic activity between aspirin-responsive patients and healthy control subjects [4, 8]. 

In summary, current theories that attempt to explain the bronchodilator effects of NSAIDs in certain individuals with asthma primarily emphasize the drugs’ impact on AA metabolism and their ability to inhibit the COX-1 pathway. However, these mechanisms cannot account for the full range of airway responses observed with NSAID use. Specifically, they do not adequately explain several key features, including the rapid onset of relief, the progressive and sustained improvement in lung function, and the consistent effectiveness of repeated aspirin administration. Given that these unique clinical responses cannot be fully explained by COX-1 inhibition alone, this hypothesis does not provide a comprehensive explanation for the bronchodilator effects experienced by this patient subset. As a result, alternative mechanisms must be considered to explain the observed improvement in respiratory function among this patient population. 

### Role of bitter taste receptors in asthma relieved by NSAIDs 

Humans can recognize five basic tastes: sour, sweet, salty, bitter, and umami [24]. Human perception of bitter taste is mediated by taste type 2 receptors (TAS2Rs), which are the expression products of the 25 human TAS2R genes [25]. TAS2Rs were first identified in taste buds on the tongue, palate, and throat and were subsequently discovered in extraoral systems, where they exert various physiological effects [26]. TAS2R agonists, such as quinine and caffeine, have been reported to trigger airway smooth muscle (ASM) relaxation [27, 28]. Studies have reported the predominant roles of TAS2R5, 10, and 14 in bitter taste agonist-induced relaxation [27]. TAS2R14, the first receptor identified to exhibit a broad agonist spectrum, has the highest expression in human bronchi among the 25 TAS2Rs [27, 29]. 

The activation of TAS2Rs triggers a complex signaling cascade via G-protein-coupled receptor (GPCR) mechanisms. Taste receptor cells are composed of several G-protein subunits, including Gα-gustducin, Gβ3, and Gγ13. In addition, the presence of multiple Gα-subunits within these cells suggests that a variety of subunits are involved in the signal transduction processes initiated by taste GPCRs [30]. 

When a bitter tastant agonist such as denatonium binds to TAS2R receptors on the surface of ASM cells, the receptors undergo a conformational change and subsequently associate with and activate α-gustducin [30]. Activation of the gustducin pathway is mediated by the release of specific gustducin subunits, Gβ3 and Gγ13, followed by activation of the enzyme phospholipase C isoform β2 (PLCβ2), which generates inositol triphosphate (IP_3_) and diacylglycerol (DAG). The newly synthesized IP_3_ binds to the inositol triphosphate receptor type III (IP_3_RIII), resulting in the release of calcium from internal stores within the sarcoplasmic reticulum of the ASM cell (Figure 1) [32]. 

The phenomenon known as the “calcium paradox” refers to the unusual observation that TAS2R activation can induce ASM relaxation despite an increase in intracellular calcium. Typically, ASM relaxation occurs as free calcium decreases in the cytosol when it is pumped out of the cell or back into the sarcoplasmic reticulum. 

This paradox highlights the need for further investigation into the specific mechanisms by which TAS2R agonists regulate ASM tone. It was proposed that bitter tastants activate large-conductance calcium- and PKC-dependent signal-activated potassium (BK) channels, thereby hyperpolarizing the membrane and leading to ASM relaxation (Figure 1) [29]. However, other studies found that bitter tastants do not activate these channels but rather inhibit them [33, 34]. Thus, more in-depth studies are required to understand how TAS2R agonists cause ASM relaxation. 

Emerging evidence highlights the critical role of gustducin βγ subunits in mediating ASM relaxation following exposure to bitter tastants. Specifically, these βγ subunits can inhibit L-type voltage-dependent calcium channels and decrease intracellular [Ca^2+^]I, which is increased by airway bronchoconstrictors, leading to airway relaxation (Figure 1) [35]. 

Recent studies have investigated the role of TAS2R in smooth muscle contraction and relaxation mechanisms. 

Regulation of smooth muscle contraction and relaxation is primarily governed by two major pathways: myosin activation and actin cytoskeletal reorganization. Most actin filaments (F-actin) are localized around myosin filaments in a rosette array, forming the contractile machinery. This pool of F-actin is referred to as “contractile actin”. In addition, “cytoskeletal actin” does not interact with myosin filaments and plays a role in maintaining the structural integrity of smooth muscle cells. The activation of myosin triggers the sliding of contractile filaments, which drives the contraction process. In parallel, the remodeling of the actin cytoskeleton facilitates the transmission of contractile forces from the filaments to the extracellular matrix, ensuring effective muscle function. Thus, myosin can be viewed to serve as an “engine” for smooth muscle contraction, whereas the actin cytoskeleton may function as a “transmission system” in smooth muscle [36]. 

Smooth muscle contraction is initiated when intracellular calcium levels are increased. The elevated calcium ions bind to calmodulin, forming a calcium-calmodulin complex. This complex activates myosin light chain kinase (MLCK), which then phosphorylates the myosin light chain (MLC). Myosin phosphorylation enables the formation of cross-bridges between F-actin and myosin filaments, allowing the contractile filaments to slide past one another and generate muscle contraction [36]. 

ASM relaxation occurs when intracellular calcium levels decline. As calcium concentration drops, myosin light chain phosphatase (MLCP) becomes active and removes the phosphate group from myosin. This dephosphorylation deactivates the cross-bridges between F-actin and myosin, leading to the cessation of filament sliding and muscle relaxation [36]. 

Cofilin also plays a pivotal role in regulating actin dynamics within smooth muscle cells. When cofilin is dephosphorylated (activated), it binds to F-actin, promoting the depolymerization and destabilization of these filaments. This interaction leads to the severing of actin filaments, which contributes to the relaxation of the actin cytoskeleton and, consequently, smooth muscle relaxation [36, 37]. 

In contrast, when cofilin is phosphorylated and thus rendered inactive, it loses its ability to bind to F-actin. This inhibition prevents the severing of actin filaments, maintaining cytoskeletal stability and supporting muscle contraction [36, 37]. 

The activity of cofilin is tightly regulated by Lim domain kinase (LIMK), an enzyme that itself becomes active upon phosphorylation. LIMK phosphorylates cofilin, thereby inhibiting its actin-severing function and promoting a contractile state in the muscle cell [36, 38]. 

Recent research suggests that TAS2R agonists can suppress RhoA signaling, which plays a crucial role in ASM contraction. Rho activation promotes F-actin polymerization in smooth muscle tissues during contractile stimulation. This suppression is achieved by modulating MLC phosphorylation. By interfering with this phosphorylation process, TAS2R agonists contribute to ASM relaxation and promote bronchodilation [36]. 

Building on these findings, a recent study demonstrated that TAS2R receptor activation can inhibit histamine-induced MLC phosphorylation. This effect is mediated by inhibition of RhoA activity and of myosin phosphatase target subunit (MYPT) phosphorylation. Together, these mechanisms further contribute to ASM relaxation and support the bronchodilator effects of TAS2R agonists [39]. 

To gain a deeper understanding of how TAS2R activation leads to bronchodilation, Woo et al. [40] conducted a study revealing the signaling cascade initiated by agonist binding to TAS2R14. They found that, upon agonist occupancy, TAS2R14 activates phospholipase Cβ (PLCβ), which subsequently interacts with partitioning defective 3 (Par3), a protein involved in establishing apical-basal polarity in epithelial cells [41]. This leads to the dephosphorylation (inhibition) of LIMK, thereby reducing its ability to phosphorylate cofilin, which thus remains in its active, dephosphorylated state. Active cofilin plays a crucial role in actin cytoskeleton remodeling by binding to filamentous F-actin and severing these filaments; this severing leads to relaxation of ASM. In addition to affecting the Par3-LIMK-cofilin pathway, PLCβ activation also increases IP3 levels. IP3 binds to its receptors on the endoplasmic or sarcoplasmic reticulum, triggering the release of calcium. This calcium release further enhances the net effect of cofilin activation, promoting ASM relaxation. In summary, the study demonstrated that the regulation of the Par3-LIMK-cofilin-actin filament axis is central to the bronchodilator effects observed following agonist activation of TAS2R14. By coordinating multiple signaling events, TAS2R14 agonists effectively promote smooth muscle relaxation and contribute to airway bronchodilation (Figure 2) [40]. 

These complex mechanisms of TAS2R14 activation result in direct, robust, and long-lasting relaxation of ASM, leading to significant bronchodilation [42]. This effect is generally more pronounced than that typically observed with conventional β-adrenergic bronchodilators [42]. 

Control of GPCR overstimulation involves receptor desensitization and downregulation [43]. Rapid desensitization occurs within minutes and involves receptor phosphorylation and the consequent uncoupling of GPCRs from their cognate heterotrimeric G proteins [43]. On the other hand, long-term desensitization occurs over hours to days and involves receptor downregulation or a decrease in cell surface receptor protein levels [43]. Attenuation of receptor responsiveness when therapeutic agonists target GPCRs may lead to a loss of effectiveness (tachyphylaxis) [43]. Use of β2-agonists for acute relief and bronchodilation in asthma leads to functional desensitization or downregulation in most patients [43]. Importantly, and in contrast to β-adrenergic agents, TAS2R14 agonists cause minimal functional desensitization and downregulation of GPCR; therefore, the bronchodilator effects can be maintained with prolonged drug use [44, 45]. 

We hypothesize that TAS2R14 plays a significant role in mediating the bronchodilation response to NSAIDs seen in certain individuals with asthma. This hypothesis arises from observations that NSAIDs can activate this receptor. Activation of TAS2R14 may help explain the rapid and effective bronchodilator response observed in some patients following NSAID administration. 

Aspirin and other NSAIDs are inherently bitter compounds. Among the various NSAIDs, most studies investigating their relationship with TAS2Rs have focused on flufenamic acid (FFA), a potent agonist and strong activator of TAS2R14 [46, 47, 48]. 

Recent research has shown that FFA binds TAS2R14 at two distinct sites: the canonical orthosteric pocket typical of GPCRs and a unique intracellular pocket. This intracellular site links TAS2R14 to its signaling partner, gustducin, thereby initiating a cascade that leads to intracellular calcium release [47]. 

Drugs that are chemically similar to FFA, such as mefenamic and niflumic acid, have also been identified as TAS2R14 agonists. Of those, only mefenamic acid has been tested in patients with asthma relieved by aspirin and exhibited a similar bronchodilator effect as other NSAIDs, such as aspirin, indomethacin, or ibuprofen [7]. 

Research has identified a noteworthy structural resemblance between TAS2R14-binding-site residues and active COX-2 sites [49], reporting that niflumic acid, mefenamic acid, and, with lower intensity, diclofenac, effectively bind to and activate TAS2R14 [50]. Due to this structural overlap, NSAIDs—which were originally developed to inhibit COX enzymes—also display significant agonist activity at the bitter TAS2R14 receptor. This dual pharmacological action stems from the shared features of the binding pockets in both the COX-2 enzyme and the TAS2R14 receptor. These observations reinforce the concept that the structural features of drug-binding sites can play a crucial role in shaping the pharmacological profiles of these compounds [48]. 

Collectively, the data reviewed offers a plausible explanation for the rapid, marked, long-lasting, and persistent bronchodilation response seen following NSAID administration in some asthma patients. 

Nevertheless, in our hypothesis, some limitations must be acknowledged. 

The primary limitation is that most research supporting the role of NSAIDs as TAS2R14 activators has focused predominantly on FFA, a compound belonging to the fenamic acid (fenamate) class of anthranilic acid derivatives, which is a nitrogen isostere of salicylic acid, the active metabolite of aspirin [49]. Other NSAIDs have received considerably less attention. For instance, only a few studies have examined indomethacin and diclofenac, indicating that, although they can stimulate TAS2R14, their effects are much weaker than those of FFA [46, 50]. The lack of comprehensive studies with a broader range of NSAIDs highlights the need for further research in this area. 

The second limitation is that the bronchodilator effect of NSAIDs is observed in only a small subset of asthmatic patients. This finding implies that these individuals possess distinct factors that enhance the efficacy of NSAIDs, setting them apart from the broader asthmatic population. 

A similar clinical scenario is evident in patients with N-ERD, who represent a relatively minor proportion of all asthma cases and are characterized by their tendency to develop bronchospasm in response to NSAID exposure [1]. Previous investigations have highlighted that patients with N-ERD present alterations in AA metabolism, which may help explain the unique clinical features observed in this group [1, 15, 17]. Drawing from these observations, it is reasonable to propose that asthma patients who experience relief from NSAIDs – particularly through mechanisms involving TAS2R14 activation – should also exhibit distinct modifications in this receptor. Such alterations could facilitate the enhanced bronchodilator response to NSAID treatment seen in this specific subset of patients. 

A substantial body of research supports the idea that genetic differences contribute to how individuals perceive all five primary taste modalities: bitter, sweet, umami, salty, and sour [51, 52, 53]. Individual genetic makeup plays a crucial role in determining how bitter taste receptors are activated [51]. One well-known example is the TAS2R38 receptor, which detects certain bitter compounds. Perceptions of bitterness for substances such as phenylthiocarbamide (PTC) vary significantly between individuals, depending on the specific combination of gene variants they harbour [52, 53]. 

Some people experience PTC as intensely bitter, while others perceive it as only somewhat bitter or even tasteless [52, 53]. This variation highlights the direct influence of genetic differences on taste receptor activation. Consequently, genetic diversity within the population leads to distinct individual responses to bitter substances [51]. Similarly, genetic differences can alter receptor function and the downstream signaling pathways of TAS2R14, thereby influencing individual responses to NSAIDs. 

Research has shown that genetic polymorphisms in the TAS2R38 receptor are a significant predictor of the progression of chronic rhinosinusitis. These variations can also influence decisions regarding treatment strategies. Specifically, reduced expression of TAS2R38 within the nasal mucosa is often indicative of advanced disease and is commonly associated with related respiratory conditions, such as asthma [54, 55, 56]. 

Studies have demonstrated that children with severe asthma exhibit elevated levels of several bitter taste receptors (TAS2Rs) compared with healthy controls. Notably, TAS2R13, TAS2R14, and TAS2R19 expressions are markedly higher in the asthmatic group. Among these receptors, TAS2R5 shows a significant correlation between its expression in leukocytes and increased bronchial hyperresponsiveness following methacholine challenge [57]. 

Functional assays utilizing bitter tastants, such as chloroquine and denatonium, have revealed that they can inhibit the release of multiple cytokines. This inhibitory effect is dose-dependent, suggesting that TAS2R activation can modulate inflammatory responses in the airways of children with asthma [57]. 

## Conclusion 

The specific mechanisms underlying asthma improvement following aspirin administration have yet to be fully elucidated. In patients who experience relief, the bronchodilator response to NSAIDs is characterized by rapid onset, significant intensity, prolonged duration, and the ability to persist with continued dosing. Our hypothesis posits that these features indicate that NSAIDs are likely to activate a receptor whose physiological effects mirror these clinical observations. Among the possible candidates, the TAS2R14 receptor best explains the clinical outcomes observed in these patients. Further supporting the hypothesis, experimental evidence demonstrates that NSAIDS such as FFA and other fenamates potently activate TAS2R14. 

However, two findings require further explanation, first the limited response of other NSAIDs such as indomethacin and diclofenac compared to fenamates and second the relatively low prevalence of asthma cases that are clinically relieved by aspirin and other NSAIDs. 

Given the substantial influence of genetic variation on TAS2R responses in the general population, we propose that specific genetic changes affecting TAS2R14 structure may partly explain the consistent NSAID response observed in this distinct subgroup of patients with asthma. Such genetic variation may help explain why only a small subset of individuals with asthma derive bronchodilator benefit from NSAIDs. 

To better understand these mechanisms, further studies should be conducted in large cohorts of asthmatic patients. Recruiting an adequate number of participants will enable assessment of TAS2R14 receptor expression in the airways, as well as facilitate genetic analyses aimed at identifying gene polymorphisms that may be associated with the bronchodilator response to NSAIDs. 

## Authors’ contributions 

CP wrote the first draft. MPT designed the figures. Both authors reviewed the final manuscript. 

## Funding 

This work did not receive any funding. 

## Conflict of interest 

Both authors declare that they have no conflict of interest. 

**Figure 1:**
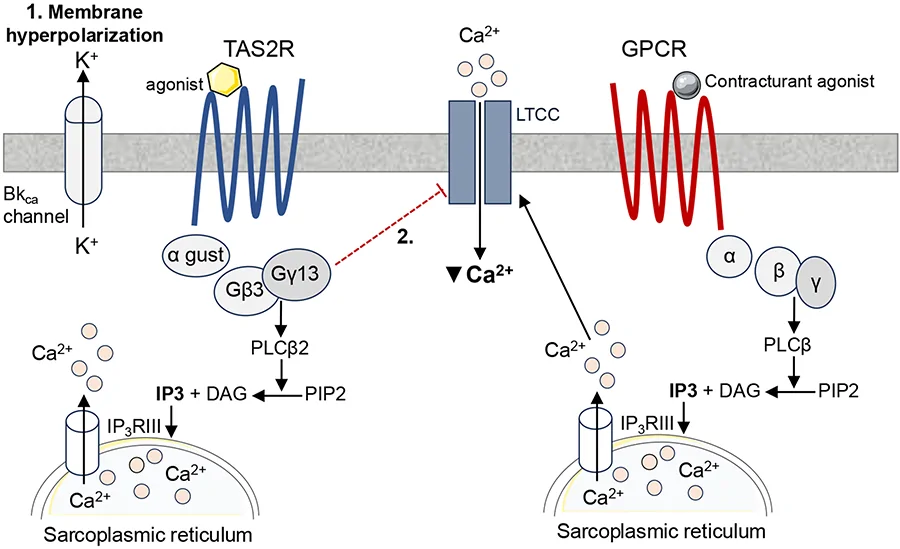
Studies suggest that TAS2R agonists relax airway smooth muscle (ASM) through several pathways. One involves signaling via TAS2R mediated by α-gustducin (α gust), and G protein subunits which mobilize activated membrane-associated phospholipase Cβ2 (PLCβ2) enzyme to cleave phospholipid phosphatidylinositol 4,5-biphosphonate (PIP_2_) into inositol 1,4,5-triphosphate (IP_3_) and diacylglycerol (DAG). This increases intracellular calcium paradoxically leading to relaxation. Elevated Ca^2+^ near the cell membrane opens potassium channels BK_ca_ causing hyperpolarization at the cellular membrane resulting in ASM relaxation (1). Another proposed mechanism is that bitter tastants counteract the Ca^2+^ increase caused by bronchoconstrictors, resulting in bronchodilation. This effect occurs through suppression of L-type voltage-dependent calcium channels via gustducin β and Gγ subunits, independent of PLCβ2 and IP3R (2).

**Figure 2:**
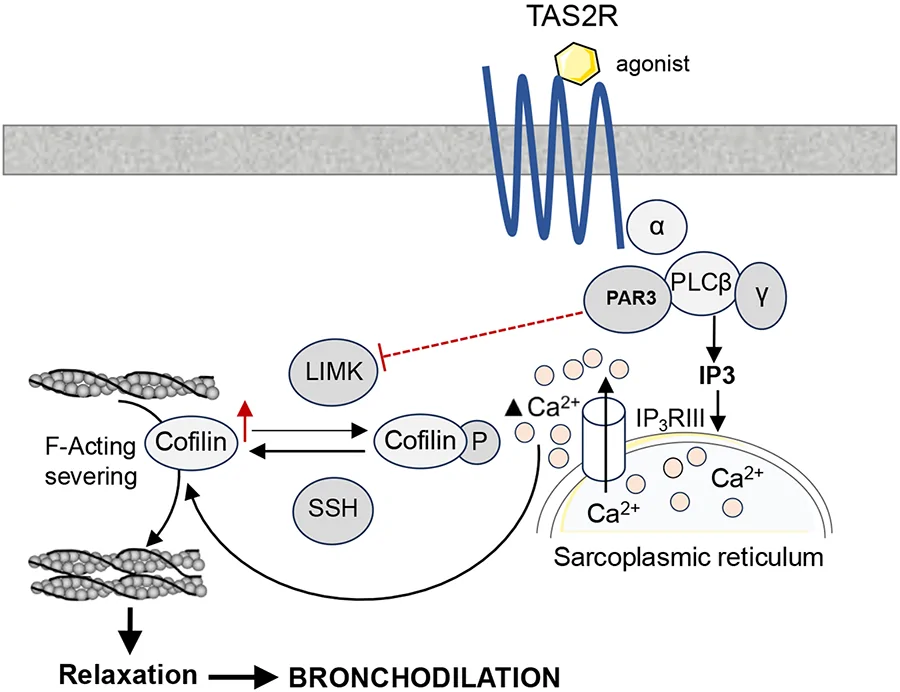
When TAS2R14 is activated by an agonist, phospholipase C beta (PLCβ) triggers partitioning-defective 3 (Par3) protein to inhibit Lim domain kinase (LIMK,) reducing cofilin phosphorylation and keeping cofilin active. Active cofilin remodels the actin cytoskeleton by severing F-actin, leading to airway smooth muscle (ASM) relaxation. PLCb also increases Inositol triphosphate (IP3), which releases Ca2+ from intracellular stores, further promoting ASM relaxation through enhanced cofilin activity. Overall, TAS2R14 agonists coordinate these pathways to relax ASM and support bronchodilation (modified from [40]).
